# Question of Quacks and Diplomas

**Published:** 1875-08

**Authors:** 


THE QUESTION OF QUACKS AND DIPLOMAS.—It has been the policy of this journal to ignore professional quarrels and difficulties, and to exclude personal matters. But there has sprung up of late an agitation which, taking its rise outside of the profession and for black-mailing purposes, has not only introduced some embarrassment in our ranks, but at the same time has tended to injure the profession in California in the estimation of persons not aware of its source and motive. Two medical periodicals, the *London Lancet* and the *Boston Journal*—no others, we believe—have permitted themselves to be duped by the publications referred to, and have committed the discourtesy of reproducing the slanders. We did not suppose that any respectable medical journalist would, without careful inquiry, propagate evil reports against his brethren separated from him by ocean and continent, on mere newspaper authority, particularly on the authority of a single publication, and that known to every individual in this quarter of the world who knows anything about it, as utterly unworthy of credence. But, as the case now stands, duty to the profession on the Pacific Coast requires us to break silence and to repel the slanders in question. This we shall do in as few words as possible, and without going far into particulars.

Some months ago, the *News Letter*, a weekly publication issued in San Francisco, began to publish, from week to week, a list of names as "quacks," including a number of respectable practitioners together with others who were most notorious charlatans. Several persons thus placarded had their names stricken from the list by paying sums of money. Other names were added, and it was announced that those who would come forward and show their diplomas should be expunged. Numbers of old and most respectable practitioners, who had graduated thirty and forty years ago, some of them in schools long since defunct, the professors all in their graves, and even the records destroyed or inaccessible, were thus publicly impeached. Many had lost their diplomas by accidents of flood and fire. Others were reasonably averse to acknowledge the authority of a libelous print to arraign them in this manner. Much excitement was aroused, in and out of the profession. Certain individuals in our ranks, who had personal ends to gain, fomented the movement by handing in the names of those whom they hated. It was well understood that there were professional scavengers engaged in this work—men who possessed diplomas and not honor. Not a solitary individual, however, up to this time, has dared to show his hand, or to acknowledge himself an informer. One or two instances of fraudulent claims to the Degree were exposed, and this gave some plausibility to the proceeding.

It was natural that men who claimed to be graduates, and who had gained membership in societies under that claim, and who were publicly charged with having no diplomas, should become subjects of inquiry among their associates. Accordingly the matter was introduced in the State Medical Society, and a committee appointed to hear and investigate charges of misconduct on that ground. So rests the question in regard to the State Society. In the San Francisco Society a proposition was made to repel the accusation as to several of its members whose names had been published in the list of quacks. The proposition was vigorously opposed by those who thought it unbecoming to notice accusations coming from such a source. The subject is under advisement, and will doubtless be disposed of properly in due season. Meanwhile, we desire to caution our brethren, both at home and abroad, not to give credence to a pretended report of the proceedings of the last meeting, which appeared in one of the daily papers, and which represented the members as having an acrimonious debate, bandying epithets, threatening resignation, and so forth. No such proceedings took place. The meeting was intended to be private, and a resolution was adopted requesting those not members of the profession to withdraw. The reporter of the daily paper alluded to, taking advantage of the fact that he was supposed to be a medical man, remained. A man who would do so, and then publish the proceedings, is destitute of honor; and one who has no honor will tell lies if it suit his purpose.

It is possible that some persons will regard our estimate of the print which originated the present turmoil as tinctured with prejudice. For the benefit of such persons, we will add that a recent number of the *News Letter* contains a long tirade of abuse and falsehood applied by name to the senior editor of this journal, which was written by a man named Neilson, a prominent attache of the former print. A number of years ago this individual was under our charge as an office patient for several weeks. He paid nothing, nor did we ever make any claim against him. Without any cause of offence, or any personal difficulty, he now publishes the studied and deliberate libel referred to—an illustration of the common experience that the beneficiaries of our profession are apt to be its worst enemies.

In conclusion, we respectfully ask the editors of the *London Lancet* and the *Boston Medical and Surgical Journal* to notice this article so far as to recall their former unfriendly publication—unless indeed they attach more credit to irresponsible and unprofessional authority on professional topics than to the declarations of responsible and well-known members of the brotherhood.—*Pacific Medical and Surgical Journal.*

Proposed Remedy for Ozœna.—A teaspoonful of common salt dissolved in a pint of milk gave "complete relief" in a case of ozœna, by persistent use as a topical application. So says Dr. Lathrop, of Detroit.

